# Targeting helminths: The expanding world of type 2 immune effector mechanisms

**DOI:** 10.1084/jem.20221381

**Published:** 2023-08-28

**Authors:** Rick M. Maizels, William C. Gause

**Affiliations:** 1 Wellcome Centre for Integrative Parasitology, School of Infection and Immunity, University of Glasgow, Glasgow, UK; 2 Center for Immunity and Inflammation, Rutgers Biomedical Health Sciences Institute for Infectious and Inflammatory Diseases, New Jersey Medical School, Rutgers Biomedical Health Sciences, Newark, NJ, USA

## Abstract

In this new review, Rick Maizels and Bill Gause summarize how type 2 immune responses combat helminth parasites through novel mechanisms, coordinating multiple innate and adaptive cell and molecular players that can eliminate infection and repair-resultant tissue damage.

## Teaser

Maizels and Gause review advances in type 2 immunity in the setting of helminth worm infections, in the skin, lung, and intestinal tract, requiring the orchestration of adaptive immunity to concentrate innate effector cells and mechanisms in scale and focus at the site of infection.

## Introduction

Type 2 immune responses underpin critical physiological processes, from protection against metazoan endo- and ectoparasites, through metabolic adaptation and homeostasis, to tissue regeneration (Gause et al., 2013); dysregulation of these responses can have pathological consequences such as allergy, impaired tissue repair, or metabolic disorders. These wide-ranging localized and systemic properties reflect the finding that both immune and nonimmune cells in the body can be recruited into the type 2 effector orbit (Inclan-Rico et al., 2022). The origins of the type 2 paradigm lie in the recognition of an adaptive immune T cell subset releasing signature cytokines (such as IL-4, IL-5, IL-9, and IL-13) acting on “professional” immune cells such as B cells and macrophages. Increasingly, however, the scope of this mode of immunity has extended to encompass a heterogeneous multiplicity of cell types that mediate immunity and repair in a highly cooperative and tissue-specific manner (Gause et al., 2020; Gieseck et al., 2018; Hammad et al., 2022; Lloyd and Snelgrove, 2018).

Type 2 immunity combines evolutionarily ancient pathways of defense and repair inherited from invertebrate systems with the more sophisticated regulation and coordination offered by the adaptive immune system. Among the forces driving the evolution of the type 2 system, the role of endoparasites (primarily helminths) and arthropod ectoparasites has been paramount. The diversity of these macroparasites and their manifold evasion strategies has demanded a corresponding diversification of defense mechanisms for host survival that are fine-tuned to each particular threat. This may explain why our picture of type 2 immunity is now far broader than originally envisaged and combines specialized sensor (afferent), expansion (adaptive), and effector (combinatorial, migratory) phases, each representing cooperation of multiple cell types and offering a high degree of redundancy (Lloyd and Snelgrove, 2018). In this review, we discuss how type 2 immunity orchestrates these effector responses, many recently discovered, that target the ancient evolutionary foe of helminth parasites.

Most attention in recent years has been given to the pathways of type 2 induction and its regulation in immunological disorders (Gause et al., 2020; Hammad and Lambrecht, 2015; McDaniel et al., 2023). In brief, the type 2 response depends on innate cell sensing of infection, invasion, or actual tissue damage, such as that resulting from large multicellular parasites trafficking through tissues and releasing degradative enzymes and other excretory/secretory products that cause cellular damage. Host innate cells respond by the release of alarmins and damage-associated molecular patterns such as IL-25, IL-33, thymic stromal lymphopoietin, uric acid, and ATP that act on myeloid cells and innate lymphoid cells (El-Naccache et al., 2021). Each of these initiates a cascade of signals to mobilize innate immunity, acting individually or in concert.

The downstream type 2 responses of innate and immune cells to helminth parasites have been well reviewed previously (Douglas et al., 2021; Grencis, 2015; Harris and Loke, 2017). Most recently, however, a set of exciting new effector mechanisms of type 2 immunity targeting helminth infections have been reported involving a diverse range of cells and mediators, including some not previously considered players in host defense. These complex pathways can be complementary, redundant, and in some cases even unique to the specific species of helminth infecting the host. Here, we review the actors involved and summarize how they are coordinated into effector consorts that mediate protective immunity through resistance and disease tolerance mechanisms in diverse tissue settings with a focus on the three key battlefields between host and parasite: the skin, the lung, and the gut.

## Immunity in the skin: Tricks and traps

Many major helminth parasites, including schistosomes and hookworms, invade the host through the skin, while others (notably *Onchocerca volvulus*, the agent of river blindness) release newborn microfilariae into the skin for onward transmission by blackfly vectors. Notably, type 2 immunity in the skin has also evolved to control ectoparasitic and hematophagous arthropods and to counter venoms from biting animals (Palm et al., 2012). Particularly in the latter setting, basophils have been implicated as the central population in protective immunity (Karasuyama et al., 2018).

**Figure 1.**
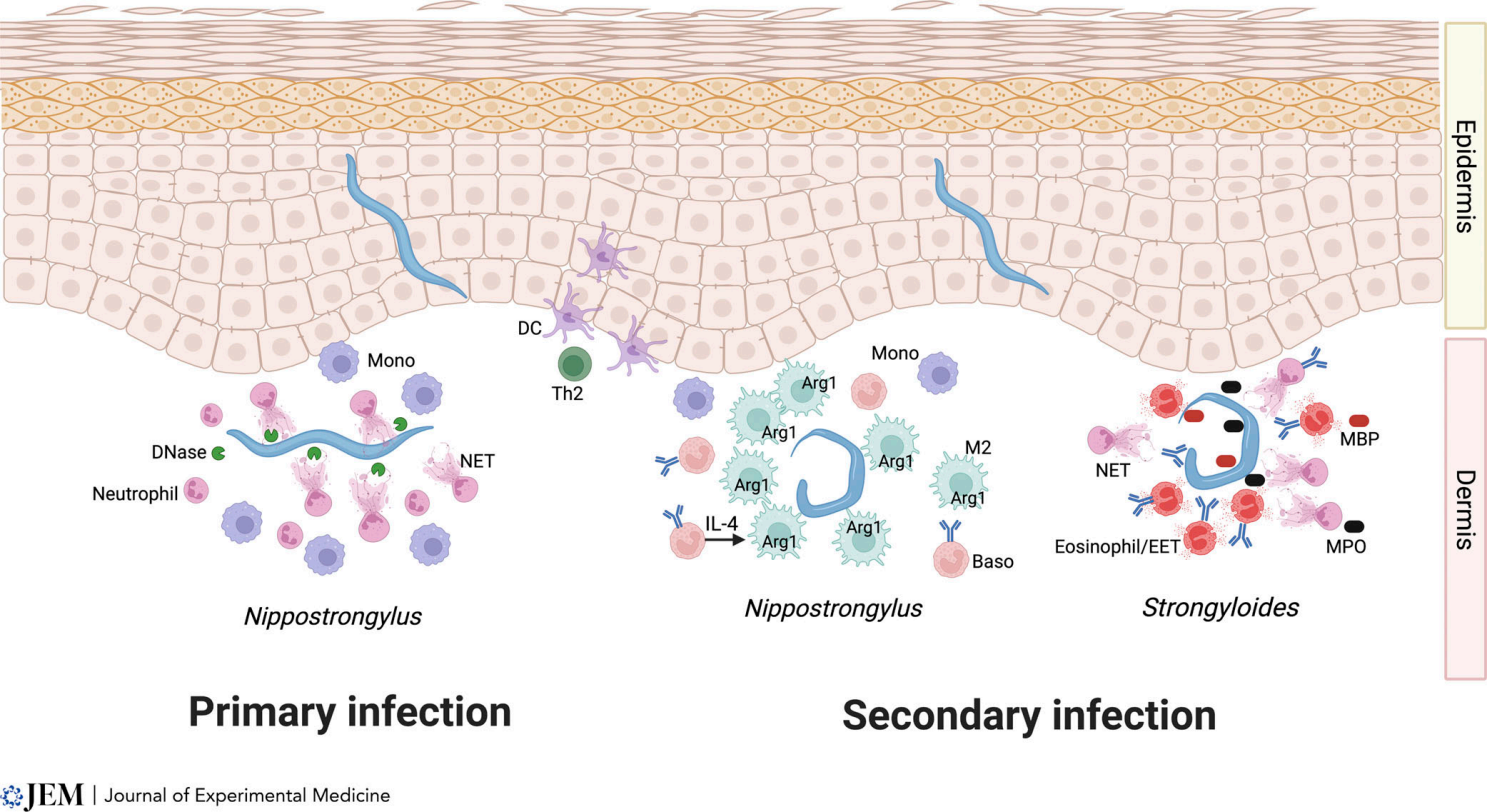
**Protective type 2 mechanisms against helminth parasites in the skin.** In primary infection (left), *N. brasiliensis* larvae are quickly attacked by neutrophils, releasing extracellular traps (NETs); however, parasite DNases can break down the NETS, allowing helminths to continue their systemic migration. In secondary infection with the same parasite (center), IgE-armed basophils release IL-4 to activate M2 macrophages (shown in green), swarming around the larvae and upregulating Arg1, which deprives parasites of an essential amino acid. Whether Arg1 actually exits the cell is not known. In infections with *S. stercoralis*, basophils are not required, but a combination of eosinophils and neutrophils can trap larvae in the skin through a combination of NETs and eosinophil extracellular traps (EETs), and the release of noxious products myelin basic protein (MBP, from eosinophils) and myeloperoxidase (MPO, from neutrophils).

During primary infection, infective hookworm larvae can escape into the bloodstream, but on re-exposure, immune mechanisms are evoked that more effectively block worm migration (Fig. 1). A good example is *Nippostrongylus brasiliensis*, which shows a similar life cycle to human hookworms, invading the skin, trafficking first to the lungs and then to the small intestine. New studies now reveal that primary infection triggers an influx of monocytes and neutrophils, with the latter making the closest contact with the larvae; this swarming behavior is followed by extrusion of neutrophil extracellular traps (NETs) that impede parasite movement (Bouchery et al., 2020; El-Naccache et al., 2020). However, in the case of *N. brasiliensis*, larval enzymes—in particular, DNase—can degrade the trap, allowing parasite migration to resume (Bouchery et al., 2020). In contrast, the immune system is more effective on secondary infection through recruitment of basophils and alternatively activated (M2) macrophages (Ohnmacht and Voehringer, 2009), with eosinophils also required for optimal immunity (Knott et al., 2007). In this setting, basophil-activated macrophages form an intense nodular reaction that immobilizes larvae in the skin and greatly reduces their migration to the small intestine. This trapping effect requires basophil production of IL-4 that activates M2 macrophages to restrain parasite migration from the skin entry point, employing Arginase-1 (Arg1)–dependent mechanisms that most likely do not involve NET formation (Obata-Ninomiya et al., 2013; Fig. 1).

It should be noted that although antibodies may not be required to mediate overall protective immunity against *N. brasiliensis*, the skin response does require IgE-armed basophils for the IL-4–mediated activation of M2 macrophages. Skin dendritic cell subsets play a critical role in initiating the antigen-specific adaptive type 2 response that dictates the outcome of infection (Connor et al., 2017; Kumamoto et al., 2013; Fig. 1). Whether host antibodies also neutralize parasite DNases in secondary infection has not been established. Interestingly, basophils are not found to play a significant role in mediating protective immunity in the small intestine, indicating that they have evolved tissue-specific functions most prominent in the skin microenvironment.

An exciting new perspective on immunity in the skin has recently been provided from studies with a related nematode parasite, *Strongyloides ratti*, a rodent model for human strongyloidiasis (Fig. 1). In this system, depletion of basophils did not compromise immune protection during secondary infection (Reitz et al., 2018); rather, skin-penetrating larvae were intercepted by eosinophils and neutrophils that prevent onward migration. Eosinophils as well as neutrophils formed extracellular traps (as reported for parasites in the pleural cavity; Ehrens et al., 2021a), and each cell type contributed key toxic molecules (major basic protein and myeloperoxidase, respectively), in a manner enhanced by specific antibody (Ehrens et al., 2021b). The demonstration of trap formation at the physiological site of infection builds on earlier work showing NET formation against the human parasite *Strongyloides stercoralis* in vitro and following intraperitoneal injection into mice (Bonne-Année et al., 2014), and fits within a recognition of the broader roles of neutrophils in immunity to helminths (Chen et al., 2014; Doolan and Bouchery, 2022). This study epitomizes the key points of immunity to helminths, in which each species is targeted by a bespoke combination of innate effector cell populations that are dependent on adaptive immune activation and the specific tissue site of invasion, ultimately deploying multiple molecular strategies to eliminate the parasite (Fig. 1). It will be interesting in future studies to elucidate the specific signals associated with different parasites triggering apparently distinct innate immune responses.

In contrast to invading nematode larvae, which attempt to rapidly transport themselves to other tissues (primarily the lung, as described below), schistosome parasites of humans remain in the skin for some days, transforming from the snail-derived cercaria to the immature mammalian stage, the schistosomulum. Strikingly, despite an overt cellular reaction in the skin, the parasites appear unscathed, in part by targeting host immune defenses through products such as Sm16 that block macrophage activation (Sanin and Mountford, 2015), and by inducing regulatory dendritic cells expressing PD-L1/2 and IL-10 (Winkel et al., 2018). In contrast, when avian-adapted schistosomes enter human skin, they are quickly trapped in an inflammatory reaction of cercarial dermatitis (colloquially termed “swimmer’s itch”; Horák et al., 2015). Given the recent demonstration of basophil activation of the itch response (Wang et al., 2021), it may be that mammalian schistosomes have evolved to neutralize cutaneous basophils to permit their survival in the skin.

Taken together, it is clear that within the skin there are multiple overlapping and non-redundant stages of protective immunity that parasites encounter as they migrate through this tissue microenvironment, and, depending on the helminth in question and the immune state of the host, they may lead to effective interception through a robust type 2 response mounted by a range of lymphoid and myeloid cells.

## Immunity in the lung: Macrophages and more

Many helminth parasites transit the lung, either en route from the skin to the intestinal tract or, as in the case of ascarid nematodes, after completing a tissue-migratory circuit that starts and finishes in the intestine. Hence, the lung is a crucial focus for immunity to helminths (Harvie et al., 2010). As with the skin, studies of *Ascaris* infection reveal eosinophil-dependent killing of lung larvae (Gazzinelli-Guimaraes et al., 2019), while the *N. brasiliensis* model indicates a pivotal role for the M2 macrophage, interacting with neutrophils and driven by type 2 cytokines. Although the M1/M2 terminology is useful, in vivo macrophages actually exhibit a spectrum of activation states and associated upregulation of key mediators (Gause et al., 2020), many of which are independently regulated and thus distinctly expressed in different milieux, as discussed below.

**Figure 2.**
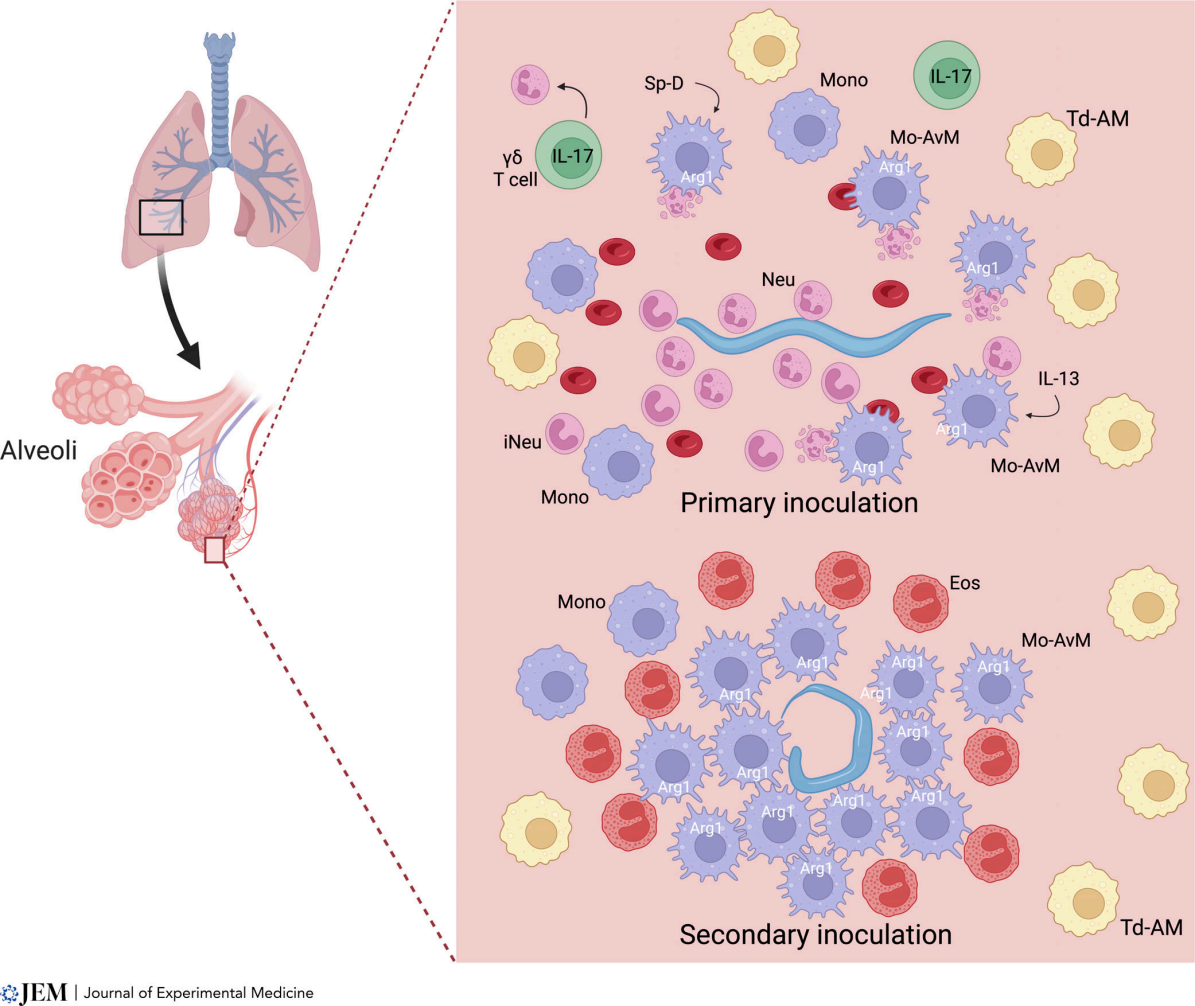
**Immune effector mechanisms in the lung.** In primary infection in the lung, an ineffective response is mobilized although it primes the immune system for effective protection to challenge infection. During *N. brasiliensis* infection, parasites transit from the skin to the lung as early as 12 h after infection and only remain in the lung for about 2 d. Associated tissue damage likely triggers γδT cells to produce IL-17 that recruit neutrophils, including an immature population (iNeu), which in turn drive M2 macrophage activation through IL-13 production and ingestion of apoptotic cells. Other signals including surfactant protein D (Sp-D) also help drive M2 macrophage activation. Neutrophils rapidly surround invading parasites, but they pass relatively unimpeded to the small intestine. Within several days after primary infection, macrophages have largely cleared RBCs and debris from the lung, and recruited monocytes have differentiated into monocyte-derived alveolar macrophages (Mo-AvMs), with an M2 phenotype. On secondary infection, migrating parasites that were able to exit the skin and invade the lung are surrounded by Mo-AvMs, which produce high levels of Arg1, depleting local arginine around the parasite, thereby mediating killing through a nutrient deprivation mechanism. Little tissue damage or neutrophil infiltration occurs and eosinophils, as well as Mo-AvMs, surround the parasite. Most parasites are killed, largely preventing their entry into the intestine.

The lung is the tissue-dwelling niche of *N. brasiliensis* larvae where they remain for 1–2 d before entering the intestine. High parasite doses cause acute lung injury (ALI), which is mediated in part by substantial IL-17–dependent neutrophil infiltration by day 2. Intriguingly, ALI is largely resolved by day 4, and this rapid lung repair is IL-4Rα dependent (Chen et al., 2012). As such, this has become a widely used model for investigating contributions of type 2 immunity to disease tolerance. An early response in the lung includes release of chitinase-like proteins that trigger TCR γ/δ cells to produce IL-17 (Sutherland et al., 2014), while surfactant protein D expression in the lung is also required for immunity to *N. brasiliensis* (Thawer et al., 2016). IL-17 recruits neutrophils (Chen et al., 2012), which are pivotal in promoting M2 macrophages (Bosurgi et al., 2017; Chen et al., 2014), and IL-17 also downregulates IFN-γ, which can contribute to the initiation of type 2 responses (Ajendra et al., 2020). Neutrophils appear to differentiate in the lung to express markers otherwise associated with M2 macrophages, including Arg1 and IL-13. These N2 neutrophils play an inductive role akin to the adaptive T helper 2 (Th2) cell in other contexts as they are essential in driving M2 macrophage activation (Bosurgi et al., 2017; Chen et al., 2014, 2022), and their depletion increases the number of parasites transiting the lung and entering the small intestine (Sutherland et al., 2014; Fig. 2).

Depletion of macrophages following infection with *N. brasiliensis* also blocks control of ALI, with M2 macrophage production of insulin-like growth factor (IGF-1; Chen et al., 2012) and resistin-like molecule α (RELMα; Krljanac et al., 2019) contributing directly to tissue repair. Clearance of apoptotic cells (Bosurgi et al., 2017) and erythrocytes through efferocytosis (Chen et al., 2012) may control harmful inflammation triggered by parasite migration, which would otherwise exacerbate tissue injury.

As discussed above, after secondary inoculation with *N. brasiliensis*, larvae are rapidly killed in the skin and lungs (Fig. 2). Interestingly, acquired resistance is intact in B cell–deficient mice (Liu et al., 2010), blocked in MHCII-deficient mice (Harvie et al., 2010), and ablated in mice treated with anti-CD4 antibody during primary infection (Bouchery et al., 2015). However, resistance is still potent in mice depleted of CD4^+^ T cells at the time of secondary infection (Bouchery et al., 2015; Katona et al., 1988), indicating that innate memory immune resistance mechanisms require CD4^+^ T cells for priming but not at the time of secondary challenge. Macrophages are known to accelerate resistance following secondary inoculation, as found when lung macrophages are transferred from *N. brasiliensis*–infected mice as late as day 45 after inoculation to naïve donors (Chen et al., 2014). Once primed in the context of infection, highly purified lung macrophages can kill larvae in vitro, first adhering to the larvae through CD11b-dependent mechanisms (Chen et al., 2014).

New studies now indicate that the mechanism of killing involves nutrient deprivation. *N. brasiliensis*–primed M2 macrophages, expressing high levels of Arg1, can rapidly deplete local arginine levels around cultured parasites and likely starve these parasites of this essential amino acid as they surround parasites in the tissue microenvironment (Chen et al., 2022; Fig. 2). Interestingly, this is analogous to the Arg1-dependent mechanism through which macrophages control inflammation in schistosome-induced granulomas, as discussed below. It will be interesting to investigate whether immune cells use other metabolic weapons against parasites, such as indoleamine 2,3-dioxygenase depletion of tryptophan or deprivation of glutamine and fatty acids taken up by M2 macrophages (Wculek et al., 2022).

In the lung, tissue-resident macrophage subsets, including tissue-derived and monocyte-derived alveolar macrophages (Td-AMs and Mo-AvMs) can play critical roles in lung homeostasis and show distinct functions and anatomical occupancy (Aegerter et al., 2022). During helminth infection, Mo-AvMs preferentially surround larvae in situ and are more effective at killing larvae in vitro, likely due to their higher expression of Arg1 (Chen et al., 2022; Fig. 2). Other recent studies have also shown that Mo-AvMs are more activated than Td-AMs following lung injury after influenza infection (Aegerter et al., 2020) or bleomycin treatment (Misharin et al., 2017).

In schistosomiasis, early studies suggested that M2 macrophages and also potentially other myeloid cells were critical in controlling harmful inflammation and associated hepatotoxicity, similar to results with IL-4Rα^−/−^ mice (Herbert et al., 2004). However, more recent studies instead suggest these myeloid cells may not be required to control susceptibility (Vannella et al., 2014). In both these studies, IL-4Rα depletion was targeted to myeloid cells with Lyz2cre, which can have incomplete penetrance. As such, new tools are needed to further investigate the functions of macrophages at this stage of schistosomiasis (Rolot and Dewals, 2018). In addition, it may be timely to revisit how immunity to lung-stage schistosomes takes place in vaccinated animals (Houlder et al., 2021).

In the chronic phase of schistosomiasis, M2-specific Arg1 expression controls type 2 inflammation and associated fibrosis in the liver, apparently by depleting local arginine levels required for sustained CD4^+^ T cell activation (Pesce et al., 2009). Differentiation of recruited monocytes to a tissue-resident phenotype in this tissue microenvironment likely plays a significant role in vitamin A–dependent macrophage maturation, control of granuloma architecture, and host survival in schistosome infection (Gundra et al., 2017).

## Intestinal helminth infection: Struggles in the submucosa

Macrophages and granulocytes are also essential in protection against helminths in the intestine, most notably when parasites invade the intestinal tissues (Anthony et al., 2007; Lechner et al., 2021). A widely used model for intestinal helminth infection is *Heligmosomoides polygyrus*, a strictly enteric natural murine pathogen (Reynolds et al., 2012). Larvae are orally ingested and rapidly cross the epithelial barrier of the small intestine, residing in the submucosal tissue for a week before developing into adults that migrate back to the intestinal lumen. Chronic infection establishes in genetically susceptible strains, but after worm clearance, a secondary infection triggers accelerated expulsion, including disrupted worm development in the tissue-dwelling phase (Liu et al., 2010).

**Figure 3.**
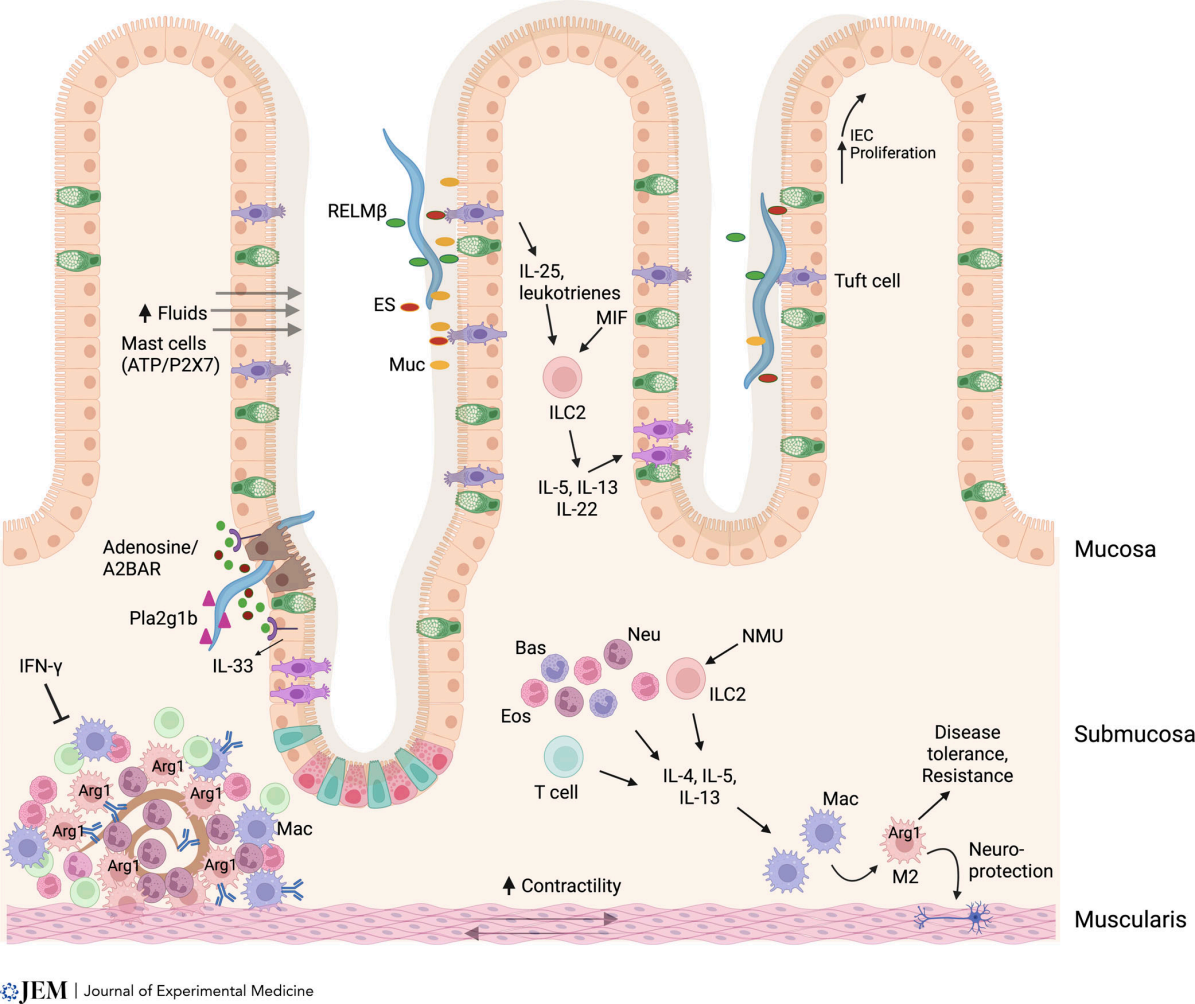
**Multiple mechanisms drive host protection to helminths in intestinal tissue.** The type 2 response is likely initiated by tuft cell sensing of helminth excretory/secretory (ES) products in the lumen. This triggers tuft cell production of leukotrienes, which, combined with constitutive tuft cell IL-25 production, and also production of macrophage migration inhibitory factor (MIF) by unknown sources and neuromedin U (NMU), induces type 2 responses. Helminths that actually invade the epithelial barrier damage intestinal epithelial cells (IECs), which release ATP. Extracellular ATP, when degraded to ADP, can bind A_2B_AR, which activate IECs contributing to tuft cell hyperplasia and IL-33 production. ATP may also bind P2X7 on mast cells. Myeloid cells and ILC2s produce type 2 cytokines driving the response. As parasites cross the intestinal barrier, group 1b phospholipase A2 (Pla2g1b) binds the parasites, impairing their development. *H. polygyrus* larvae dwell in the submucosa as they develop into adults. After secondary inoculation, a rapidly developing granuloma, composed primarily of M2 macrophages, and also eosinophils, surrounds the parasite and impairs its development through Arg1-dependent mechanisms. Type 2 cytokines drive the type 2 granulomas while IFN-γ suppresses it. Helminths in the lumen may be expulsed through a combination of fluids flowing into the lumen, enhanced by activated mast cells, production of mucins (muc), and increased muscle contractility (weep-and-sweep response). Also, parasites, such as *Trichuris*, residing in the epithelial layer may be expulsed through upward IEC proliferation. M2 macrophages also mediate enteric disease tolerance mechanisms including neuronal protection.

After migrating to the submucosa during a secondary inoculation, *H. polygyrus* larvae are rapidly surrounded by M2 macrophages, neutrophils, and eosinophils, forming a dense type 2 granuloma, which is also CD4^+^ T cell dependent (Anthony et al., 2006; Hewitson et al., 2015; Fig. 3). M2 macrophages, which play an essential role in mediating acquired resistance during the tissue-dwelling phase, inhibit parasite development through IgG1- and Arg-1–dependent mechanisms (Anthony et al., 2006; Esser-von Bieren et al., 2013; Hewitson et al., 2015). They also produce quantities of chitinases and RELMs that play regulatory roles, with acidic mammalian chitinase required to amplify the type 2 protective response to intestinal nematodes (Vannella et al., 2016), while RELMα restrains type 2–mediated pathology (Chen et al., 2016). Trapping of larvae within the granuloma is enhanced by an epithelial cell product, group 1B phospholipase A2, which directly impairs larval development and is produced on stimulation by CD4^+^ T cells (Entwistle et al., 2017; Fig. 3).

The granulomatous response to *H. polygyrus* is most intense in mouse strains (such as SJL), mounting the most rapid Th2 response to infection (Filbey et al., 2014), while susceptible strains (such as C57BL/6) develop few granulomas during primary infection. However, in the absence of classical inflammatory signals mediated by MyD88, IL-1R1, or IFNAR, significant granuloma formation occurs in primary infection on the C57BL/6 background (Reynolds et al., 2014), as also recently reported in mice carrying an enteric glial cell–specific conditional deletion of the IFN-γ receptor (Progatzky et al., 2021). A more detailed analysis of the molecular pathways that promote or inhibit type 2 granuloma formation would be most illuminating.

New studies further indicate that eosinophil production of IL-4 and IL-13 activate M2 macrophages in the muscularis, enhancing disease tolerance, including neuronal protection, after primary intestinal infections with *H. polygyrus* and other helminth parasites (Ahrends et al., 2021), which would be consistent with their newly recognized role as mediators of homeostasis in the gut (Ignacio et al., 2022). In reciprocation, Arg-1 from macrophages in the muscularis also acts to protect the enteric nervous system from damage (Ahrends et al., 2021; Matheis et al., 2020; Fig. 3).

Indeed, several studies have established multiple levels of neuroimmune dialogue, which can both positively or negatively impact helminth immunity, including production of neuromedin U that activates type 2 innate lymphoid cells (ILC2s) in infection to promote immunity (Cardoso et al., 2017; Klose et al., 2017), together with the negative effects of adrenergic signaling on ILC2s (Moriyama et al., 2018), and the involvement of enteric glial cells that produce IFN-γ during *H. polygyrus* infection that counteracts the formation of granulomas around tissue larvae (Progatzky et al., 2021). It remains to be determined whether IFN-γ is stimulated by bacterial translocation or by parasite tissue disruption.

Several helminth infections are strongly associated with mast cell outgrowth, and pharmacological mediators from mast cells may impact both epithelial permeability (“weep”) and smooth muscle activity (“sweep”) discussed below. In the case of *H. polygyrus*, mast cell–deficient mice are more susceptible to both primary and challenge infection (Hepworth et al., 2012), while mice lacking the transcription factor Spib (which is required for intestinal M cell differentiation and contributes to plasmacytoid dendritic cell development) were found to have greatly expanded mast cell numbers and to show a high level of resistance to *H. polygyrus*. Interestingly, mast cell expansion was dependent on the purinergic receptor P2X7, which responds to extracellular ATP, although in WT mice blockade of P2X7 did not affect worm burden (Shimokawa et al., 2017).

## Helminths in the lumen: A multicellular response

In secondary infection, *H. polygyrus* adults that do reach the intestinal lumen are likely weakened and are rapidly expulsed through a combination of increased luminal fluids, muscle contractility, and mucus secretion (Coakley and Harris, 2020). This well-characterized “weep-and-sweep” response includes such classical responses as heightened barrier permeability and mucus production (Hasnain et al., 2013; McKay et al., 2017), epithelial proliferation driven by IL-13 and amphiregulin (Cliffe et al., 2005; Zaiss et al., 2006), and smooth muscle hypercontractility dependent on M2 macrophages (Zhao et al., 2008). More recently, several exciting developments have moved this field forward as discussed below—first, by revealing mechanistic detail, and second, by uncovering interconnectedness, particularly with respect to the enteric nervous system.

Tuft cells are a key cell type in the intestinal epithelium that constitutively produce IL-25. While uncommon in the steady-state, they are both activated and expanded in helminth infection: activation drives release of cysteine leukotrienes, priming ILC2s, which trigger increased differentiation of new tuft cells and associated increases in IL-25 in a feed-forward amplification loop (Gerbe et al., 2016; Schneider et al., 2019) that requires both IL-25 and the pleiotropic cytokine macrophage migration inhibitory factor (Varyani et al., 2022). However, tuft cell–deficient mice are more susceptible to *N. brasiliensis* infection than animals lacking IL-25, and their marked expansion after a type 2 response has been launched argues that their function may extend beyond detection to participating in expulsion. Notably, they produce a range of other small molecule mediators, including leukotrienes. However, in the absence of Alox5, *N*. *brasiliensis* expulsion is substantially delayed but not prevented, indicating compensating or redundant mechanisms (McGinty et al., 2020). Tuft cells also produce acetylcholine, as do innate lymphoid cells, which in *N. brasiliensis* is required for optimal clearance (Chu et al., 2021; Roberts et al., 2021). Interestingly, recent studies indicate that epithelial cell elevations in Alox5 and tuft cell hyperplasia are dependent on adenosine binding the A_2B_ adenosine receptor (A_2B_AR), providing a potential additional signal for tuft cell activation (El-Naccache et al., 2022).

*H. polygyrus* interferes with arachidonic acid metabolism, switching from Alox to COX, forming PGE2, and blocking the type 2 response (de Los Reyes Jiménez et al., 2020). However, the role of such eicosanoids in intestinal worm infection is not always intuitive, as PGD2 and its receptor CRTH2 surprisingly inhibit the type 2 epithelial response resulting in greater susceptibility to *N. brasiliensis* (Oyesola et al., 2021).

Goblet cells are classically associated with gastrointestinal helminth infections, displaying hyperplasia in both frequency and morphology in response to IL-4/-13 signaling via STAT6 (Khan et al., 2001) and producing high levels of mucins and RELM-β with direct anti-helminth properties (Artis et al., 2004; Herbert et al., 2009). Most recently, an IL-13–independent route promoting goblet cell differentiation, driven by IL-22, has been discovered, which preferentially induces RELM-β (Lindholm et al., 2022). As IL-13–driven mucin expression is important for resistance (Hasnain et al., 2013), while IL-22–deficient mice are more susceptible to both *N. brasiliensis* and *Trichuris muris* (Turner et al., 2013), both goblet cell phenotypes may be necessary for optimal anti-helminth immunity.

The intimate involvement of the nervous system in immunity is nowhere more conspicuous than in inducible hypercontractility of smooth muscle cells that is driven both by immune cytokines (IL-13 and IL-25 in particular) and neurotransmitters such as acetylcholine and serotonin (Zhao et al., 2006). Hypercontractility is dependent on M2 tissue macrophages (Zhao et al., 2008), although it is not yet clear whether they are extrinsic to the muscle tissue or are intrinsic muscularis macrophages that are strongly anti-inflammatory in function (Ahrends et al., 2021). As there is also an intimate relationship between mast cells and the gut-nervous system (Jacobson et al., 2021), further analysis of these interactions would be fruitful.

In nearly all settings, antibodies play an important, if not indispensable role in host immunity to helminths (Zaini et al., 2021). As discussed above, they may arm basophils in the skin to release IL-4 for protective macrophages (Obata-Ninomiya et al., 2013), trap helminth larvae in the tissues (Esser-von Bieren et al., 2013), activate myeloid cells (Esser-von Bieren et al., 2015), and neutralize parasite products that interfere with host immunity (Hewitson et al., 2015).

Protective immune responses do not, of course, go unchallenged by the parasites themselves. Some, such as *H. polygyrus,* induce immune-suppressive regulatory T cells (Tregs) which control susceptibility (Smith et al., 2016) or block the expansion of epithelial goblet and tuft cells to prevent expulsion (Drurey et al., 2022; Karo-Atar et al., 2022). Even the alarmin IL-33 plays contrasting roles in different settings, amplifying Th2 immunity when released from the intestinal epithelium, but promoting Tregs when expressed by dendritic cells in infection (Hung et al., 2020).

## Aftermath: Repair and return to homeostasis

The immune system functions to help maintain and restore homeostasis through repair pathways in different tissues, such as pulmonary macrophages producing IGF-1 and RELMα (Chen et al., 2012; Gause et al., 2013; Gieseck et al., 2018), or fibro/adipocyte precursors responding to eosinophil-derived IL-4 in the case of injury to muscle tissue (Heredia et al., 2013). However, the state of homeostasis is dynamic, adapting to environmental exposures to pathogens and other insults. Following infection, the system recalibrates to an altered steady state that combines conventional immunological memory as well as trained innate immunity (Hartung and Esser-von Bieren, 2022), a distinction that is increasingly blurred (Guo et al., 2015).

Long-term tissue remodeling can result from even transitory helminth infection. In the lung, a chronically altered lung tissue environment includes a persistent M2 macrophage phenotype, which not only has anti-helminth properties, as discussed earlier, but also protects against infection with SARS-Cov-2, even as late as 28 d after *N. brasiliensis* inoculation (Oyesola et al., 2023). However, the remodeling can also take an aberrant course, as observed in the IL-17–dependent emphysema that is severe by 30 d after *N. brasiliensis* infection (Chen et al., 2018; Marsland et al., 2008), even though the parasite resides in the lung for only 2–3 d. Interestingly, RELMα production by B cells 1–2 d after infection controls IL-17 elevations, delaying development of emphysematous pathology (Chen et al., 2018).

In studies with the strictly enteric pathogen *H. polygyrus*, muscularis M2 macrophages acquire a tissue protective phenotype that maintains enteric nervous system functionality following infection with heterologous pathogens even in separate regions of the intestine (Ahrends et al., 2021). Neuroprotection also occurs during the intestinal response to *Trichinella spiralis*, where monocytes recruited to the brain express M2 markers and inhibit excessive microglial cell activation. This tolerance mechanism persists for months and also protects against lipopolysaccharide-induced neuroinflammation (Peng et al., 2022).

Following *H. polygyrus* infection, the distal skin CD4^+^ T cell composition becomes Th2 skewed with impaired IFN-γ recall responses to *Mycobacterium tuberculosis* antigen months after deworming treatment (Classon et al., 2022). Distal tissue responses to infection with the same parasite have also been observed in the lung where responses to allergens are mitigated by Treg cells (Wilson et al., 2005), while in *N. brasiliensis* infection, lung and small intestinal ILC2s disperse widely to underpin a systemic type 2 response (Huang et al., 2018; Ricardo-Gonzalez et al., 2020).

In each of these different models, it remains to be determined if the phenotypes of specialized protective cells can persist autonomously, potentially through epigenetic changes, or are instead sustained by other immune and nonimmune components of the conditioned tissue microenvironment remains unclear.

In the case of CD4^+^ T cells at least, persistent T cell responses develop in the peritoneal cavity, where memory Th2 cells can rapidly support parasite-specific recall responses (Steinfelder et al., 2017; Yordanova et al., 2022). In other studies, persistent Th2 cells have been identified in the mesenteric adipose tissue after *H. polygyrus* inoculation, retained for at least 11 mo after worm clearance (Kabat et al., 2022). Furthermore, these long-lived tissue Th2 cells act in many respects like innate lymphocytes, sustained by stromal cell responses. Similarly, Th2 cells produce IL-13 in vivo following TCR-independent stimulation with a combination of IL-33 and a STAT5 activator, such as thymic stromal lymphopoietin; indeed, these cells, as opposed to ILC-2s, may comprise the major source of Th2 cytokines in the antigen-experienced host that can even prove protective against primary *N. brasiliensis* infection (Guo et al., 2015).

Thus, helminth infection remodels host tissue architecture and associated immune responses even at sites distal to infection and favors the emergence of antigen-independent T cell activation. These changes are protracted and reset the level of homeostasis, resulting in long-term changes in tissue architecture and function and influencing immune responses to subsequent homologous and heterologous infections, demonstrating that “trained immunity” can be a major factor in helminth infections (Hartung and Esser-von Bieren, 2022). Especially important in the context of an altered steady-state following exposure to helminth antigens, hematopoietic stem cells in the bone marrow switch to favor anti-inflammatory Ly6C(lo) macrophage differentiation (Cunningham et al., 2021), while evidence is accumulating that maternal helminth infection can imprint immune responsiveness in human offspring (Lacorcia and Prazeres da Costa, 2018). Overall, such effects of prior helminth infection may potentially enhance host protective responses against homologous or heterologous pathogens or, in other settings, exacerbate disease susceptibility (Salgame et al., 2013). Although this aftermath of helminth infection has significant real-world global health significance, the associated mechanisms remain little studied.

## Conclusion

Type 2 immunity, like the helminth parasites it targets, touches every tissue in the body and assembles complex consortia of cells and mediators in each different context. The specific effector functions contributing to protective immunity depend greatly on the parasite in question and its tissue microenvironment and involve multiple redundant pathways to deal with varied parasite evasion mechanisms. Overall, our understanding of how the type 2 immune response mediates effector functions in different tissue microenvironments is still poorly developed. Such understanding is now needed for the development of next-generation vaccines and therapies against helminth infection, and may also inform new strategies to ameliorate type 2–mediated disorders such as asthma, allergic dermatitis, and ulcerative colitis.

As type 2 immune responses and associated effector cell interactions vary greatly with the type of invading helminth and its environment, custom-tailored strategies may be required to maximize responses. In this context, a number of reports now show a variety of myeloid and lymphoid cells make type 2 cytokines in barrier environments, and it will be important in future studies to distinguish when these play essential, additive, or redundant roles. Increasingly, the development of innate memory responses is recognized as a critical component of acquired resistance. Teasing apart the contribution of extrinsic signals likely provided by chronic tissue-specific remodeling, involving immune and nonimmune compartments, and intrinsic modifications, including long-term epigenetic changes in effector cells and their progenitors, is needed to identify potential checkpoints that can be targeted to enhance these responses. How this tissue-specific immune cell reprogramming affects the course of subsequent heterologous infections may provide a deeper understanding of the heterogeneity of individual responses to pathogens generally.

At the same time, the profound nature of protective mechanisms against helminths (forming major lesions, compromising nutrition, consuming energy) requires much greater regulation and subjection to the evolutionary compromise of “disease tolerance” (Medzhitov et al., 2012). Finally, while type 2 may be the ideal tissue immunity, it is not always effective in either eliminating pathogens or forestalling pathology; a greater understanding of its complexity and how to direct it most favorably is required to address the major health problems of parasitic helminth infections.
